# Clinical Characteristics and Influenza-Associated Myositis in Children: A Comparative Study of Influenza A and Influenza B in Bahrain

**DOI:** 10.7759/cureus.114703

**Published:** 2026-08-17

**Authors:** Abdulrahman D Mohroofi, Mohamed Alawadhi, Fatima K Alqanea, Gabriel Fox

**Affiliations:** 1 Medicine, Arabian Gulf University, Manama, BHR; 2 Pediatrics, King Hamad University Hospital, Manama, BHR

**Keywords:** bahrain, benign acute childhood myositis, creatine kinase, influenza a, influenza b, pediatric influenza, viral myositis

## Abstract

Background and objective

Influenza-associated myositis is an uncommon but increasingly recognized complication of pediatric influenza infection. Although influenza B has traditionally been associated with benign acute childhood myositis (BACM), data comparing the clinical characteristics of influenza A and influenza B in children with influenza-associated myositis remain limited, particularly in the Middle East. Hence, this study aimed to compare the clinical presentation, laboratory findings, outcomes, and prevalence of myositis-related manifestations among children with influenza A and influenza B infections.

Methods

A retrospective cross-sectional study was conducted at King Hamad University Hospital, Bahrain. Medical records of pediatric patients aged ≤ 14 years with confirmed influenza A or influenza B infection between October 2023 and January 2024 were reviewed. Demographic characteristics, clinical features, laboratory findings, hospitalization data, treatments, and complications were analyzed and compared between influenza A and influenza B groups.

Results

Ninety-six children with laboratory-confirmed influenza infection were included. Influenza B was associated with a significantly higher frequency of calf pain (n = 8, 25.8% versus n = 5, 7.7% in influenza A cases; p = 0.015) and refusal to walk, reported in seven children (22.6%) compared with five children (7.7%) with influenza A (p = 0.039). No significant differences were identified between the two groups regarding laboratory findings, hospitalization requirements, duration of admission, or complication rates. Markedly elevated creatine kinase (CK) levels were observed in both influenza subtypes, consistent with influenza-associated muscle injury.

Conclusions

Influenza B infection was associated with a significantly higher frequency of myositis-related manifestations, including calf pain and refusal to walk, compared with influenza A infection. Despite these clinical differences, laboratory findings, hospitalization rates, and clinical outcomes were comparable between the two groups. Recognition of influenza-associated myositis is essential when evaluating children with acute lower-limb pain or gait disturbances during influenza season, as early diagnosis may help avoid unnecessary investigations and support appropriate management.

## Introduction

Influenza is a contagious viral infection that primarily affects the respiratory system. Influenza infections are most prevalent during seasonal outbreaks, with incidence varying by season and generally increasing during the winter and early spring months [1]. While influenza typically presents with respiratory symptoms, such as fever, cough, sore throat, and nasal congestion, the infection has also been associated with myositis, which usually manifests approximately three days after resolution of the initial viral illness [2].

Myositis is characterized by inflammation of the muscles, resulting in pain, weakness, and swelling in the affected muscle groups. The mechanisms underlying the development of myositis in children with influenza infection are not fully understood. Influenza-associated myositis typically presents with muscle pain and weakness, predominantly affecting the calf and thigh muscles [3]. Marked elevations in creatine kinase (CK) levels, often reaching thousands of units, are frequently observed and are typically accompanied by normal inflammatory markers, mild leukopenia, and thrombocytopenia [4]. Gait abnormalities have been reported as the initial presentation to the emergency department in 77% of cases [5].

Data on influenza-associated myositis cases remain scarce, with few reports available from the Arabian Gulf region and the Middle East [2,6]. In Bahrain, no studies have been published on this topic, and only two studies have addressed the epidemiology of influenza infection [7,8]. This study aimed to assess differences in the clinical presentation and laboratory findings between influenza A and B infections, as well as the prevalence of influenza-associated myositis. We believe that this research can contribute to the development of clinical guidelines to help healthcare professionals recognize differences between influenza types, as well as the expected outcomes and complications associated with influenza infection. Improved recognition may facilitate appropriate treatment, potentially reduce the severity of complications, and minimize unnecessary interventions.

## Materials and methods

Study design and setting

This was a retrospective cross-sectional study in which electronic medical records of all patients who presented to the pediatric emergency department of King Hamad University Hospital with a positive influenza A or B result between October 2023 and January 2024 were retrieved. Patients were excluded if they tested positive for other viruses, had an identifiable alternative cause of lower-limb weakness, such as peripheral neuropathy, Guillain-Barré syndrome, multiple sclerosis, or poliomyelitis, or were older than 14 years.

Data collection

Influenza A and B infections were detected using the Cepheid Xpert® real-time PCR panel (Cepheid, Sunnyvale, CA), and test results were obtained from the hospital laboratory database with assistance from laboratory personnel. Demographic data (age, sex), clinical symptoms (cough, rhinorrhea, sore throat, myalgia, muscle weakness, etc.), and laboratory findings (complete blood count, C-reactive protein, and CK) were recorded for each patient. The duration of hospital stay, clinical outcomes, and need for medications were also documented. All eligible patients during the study period were included, and data were systematically recorded in a standardized format to ensure consistency. Ethical approval was obtained from the King Hamad University Hospital Research Ethics Committee.

Statistical analysis

Statistical analysis was performed using IBM SPSS Statistics for Windows, Version 25.0 (IBM Corp., Armonk, NY). Data normality was assessed using the Shapiro-Wilk test. Categorical variables were compared using Pearson's chi-square test or Fisher's exact test, as appropriate, while continuous variables were compared using the independent-samples t-test or Mann-Whitney U test, depending on the data distribution. A two-sided p-value < 0.05 was considered statistically significant. No formal sample size calculation was performed because the study was retrospective and observational. All eligible pediatric patients who met the inclusion criteria during the study period were included; therefore, the sample size was determined by the total number of eligible cases available during the study period.

## Results

Demographic data and clinical presentation

**Figure 1:**
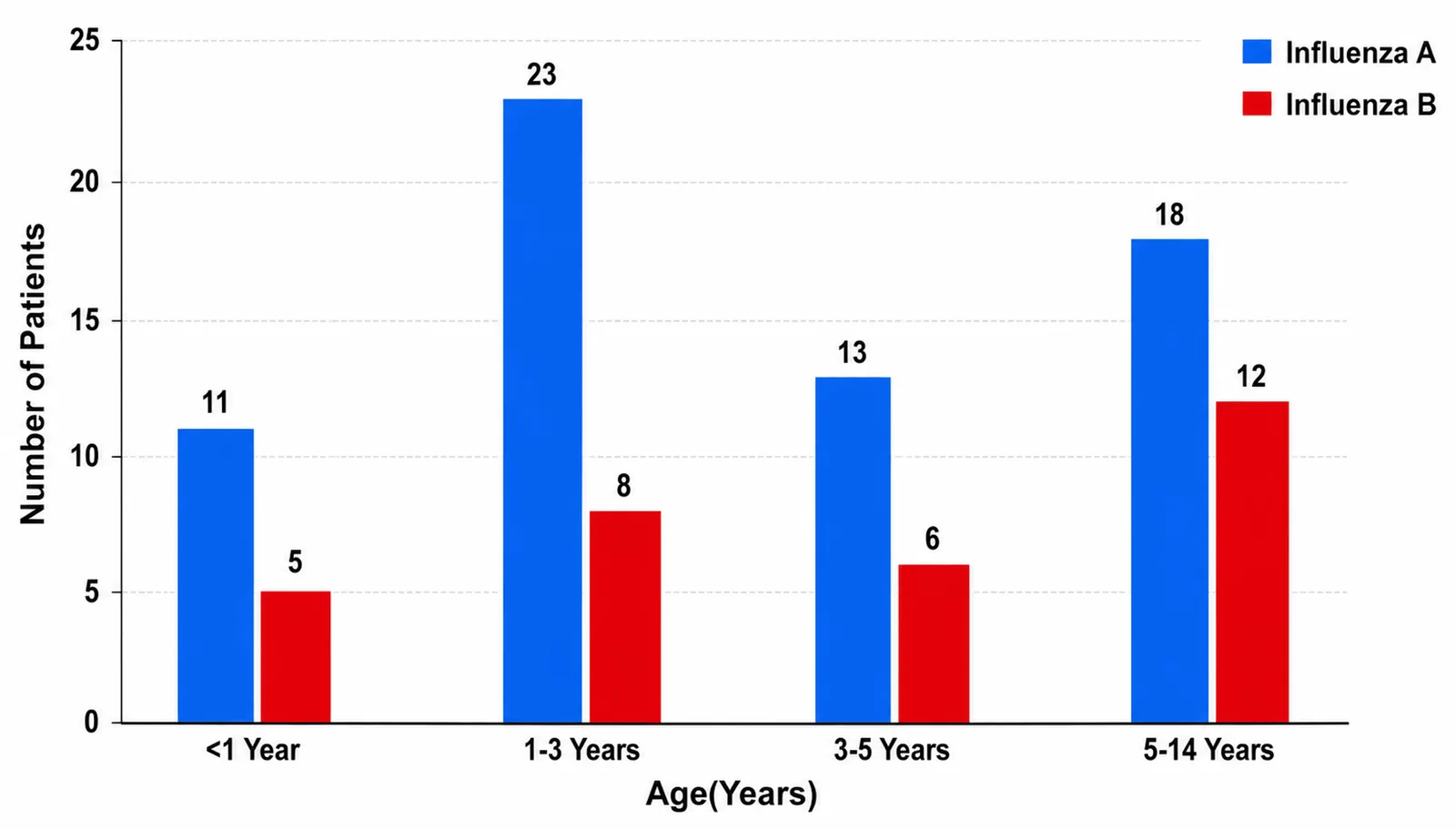
Age distribution of children with influenza A and influenza B

A total of 96 pediatric patients with confirmed influenza A or influenza B met the inclusion criteria during the study period. The mean age was 4.15 years (SD: 3.40), and 57 patients (59.4%) were male (Figure 1).

**Figure 2:**
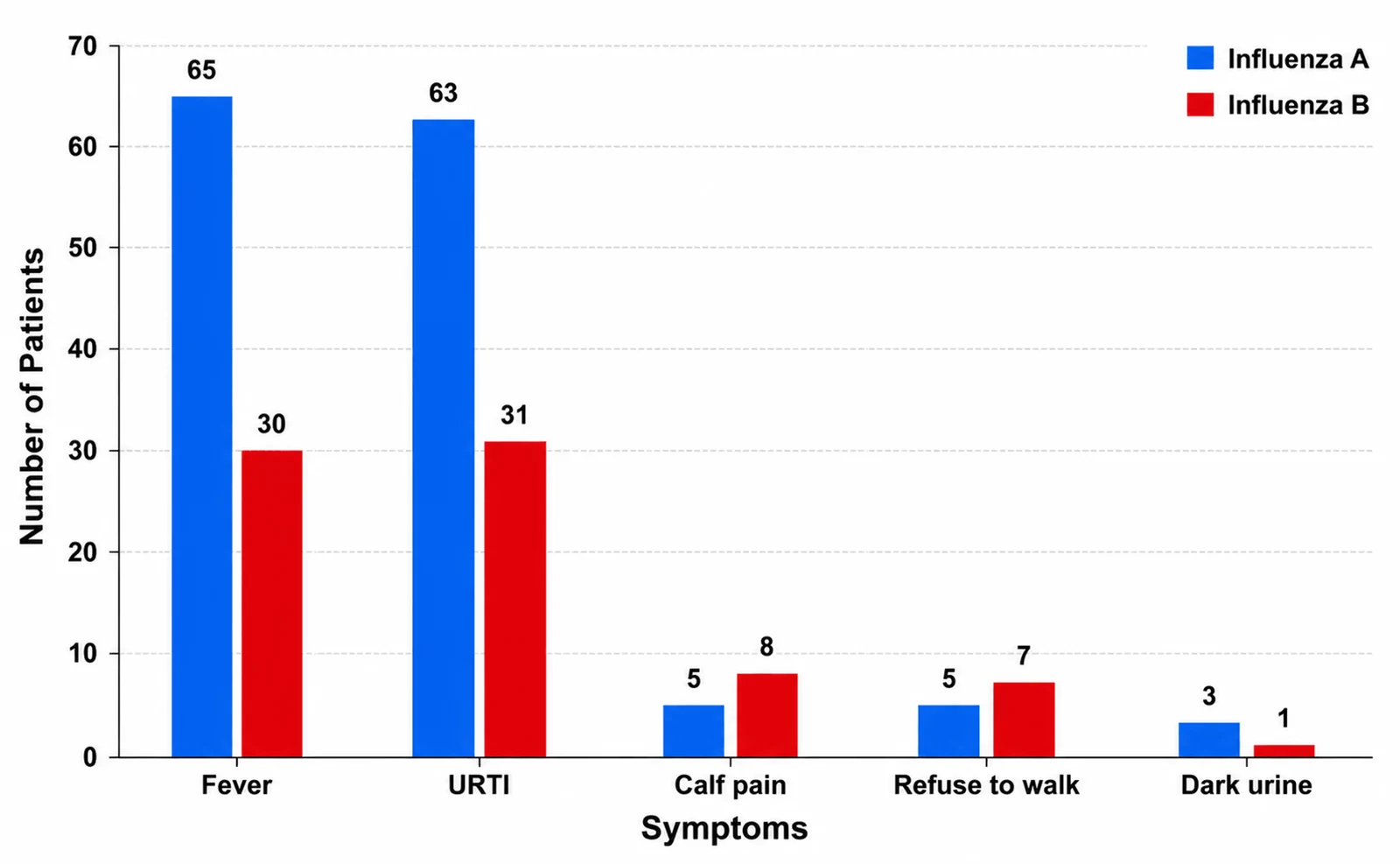
Symptoms across influenza A and influenza B

Influenza A was identified in 65 children (67.7%), whereas 31 children (32.3%) had influenza B. Fever and upper respiratory tract infection symptoms were the most common presenting features, reported in 95 patients (99.0%) and 94 patients (97.9%), respectively, reflecting the typical manifestations of influenza infection in children. Calf pain was reported in 13 patients (13.5%) and refusal to walk in 12 patients (12.5%), while dark urine was documented in four patients (4.2%) (Figure 2).

Muscle-related symptoms were significantly more prevalent in children infected with influenza B compared to those infected with influenza A. Specifically, calf pain occurred in eight children (25.8%) with influenza B compared with five children (7.7%) with influenza A (p = 0.015), and refusal to walk was reported in seven children (22.6%) with influenza B compared to five children (7.7%) with influenza A (p = 0.039). These findings highlight a notable difference in the clinical presentation of viral myositis symptoms between the two influenza types in pediatric patients. In contrast, no significant differences were observed between influenza A and B regarding gender distribution (p = 0.532), fever occurrence (p = 0.145), upper respiratory tract infection symptoms (p = 0.324), or the presence of dark urine (p = 0.578). These results suggest that general demographic characteristics and common flu symptoms are similarly distributed across both influenza types in the pediatric population (Table 1).

**Table 1:** Comparison of demographic and clinical characteristics between influenza A and influenza B Comparisons between influenza A and influenza B were performed using Pearson's Chi-square test when its assumptions were met; otherwise, Fisher's exact test was used. Statistical significance was defined as a two-sided p < 0.05. Two patients with missing data for dark urine (both in the influenza A group) were excluded from the analysis of this variable URTI: upper respiratory tract infection

Variable	Category	Influenza A (n = 65), n (%)	Influenza B (n = 31), n (%)	Total (n = 96), n (%)	Test statistic	P-value
Gender	Male	40 (61.5 %)	17 (54.8%)	57 (59.4%)	x^2^ = 0.391	0.532
	Female	25 (38.5 %)	14 (45.2%)	39 (40.6%)		
Fever	Yes	65 (100%)	30 (96.8%)	95 (99%)	Fisher’s exact test	0.323
	No	0 (0%)	1 (3.2%)	1 (1%)		
URTI	Yes	63 (96.9%)	31 (100%)	94 (97.9%)	Fisher’s exact test	1.000
	No	2 (3.1%)	0 (0%)	2 (2.1%)		
Calf pain	Yes	5 (7.7%)	8 (25.8%)	13 (13.5%)	Fisher’s exact test	0.024
	No	60 (92.3%)	23 (74.2%)	83 (86.5%)		
Refusal to walk	Yes	5 (7.7%)	7 (22.6%)	12 (12.5%)	Fisher’s exact test	0.051
	No	60 (92.3%)	24 (77.4%)	84 (87.5%)		
Dark urine	Yes	3 (4.8%)	1 (3.2%)	4 (4.3%)	Fisher’s exact test	1.000
	No	60 (95.2%)	30 (96.8%)	90 (95.7%)		

Laboratory findings

Comparison of laboratory findings between patients diagnosed with influenza A (n = 65) and influenza B (n = 31) was performed, as presented in Table 2. The results showed no statistically significant differences between the two groups across all measured parameters, with all p-values exceeding 0.05. Overall, the laboratory profiles did not differ significantly between patients with influenza A and influenza B infections.

**Table 2:** Comparison of demographic and laboratory parameters between influenza A and influenza B Test statistics are presented as the independent samples t-test (t) or Mann–Whitney U statistic (U), as appropriate. A two-sided p-value < 0.05 was considered statistically significant SD: standard deviation; IQR: interquartile range

Variable	Influenza A (n = 65)	Influenza B (n = 31)	Test statistic	P-value
Age (years)	3 (1-6)	5 (1-7)	U = 897.50	0.387
White blood count, × 10³/µL, median (IQR)	6.9 (4.7-9.8)	6.7 (4.6-11.1)	U = 685.50	0.668
Platelets, × 10³/μL, median (IQR)	231.0 (170.0-312.0)	249.0 (186.0-325.0)	U = 658.50	0.753
Aspartate aminotransferase, U/L, median (IQR)	37.7 (27.9-65.2)	59.3 (34.6-148.4)	U = 110.50	0.158
Alanine aminotransferase, U/L, median (IQR)	15.1 (13.1-27.5)	23.0 (15.9-48.0)	U = 72.00	0.084
Gamma-glutamyl transferase, U/L, median (IQR)	11.8 (9.9-13.9)	13.0 (8.9-98.8)	U = 108.00	0.935
Alkaline phosphatase, IU/L, mean ± SD	195.9 ± 66.9	187.7 ± 62.9	t = 0.325	0.748
Urea, mmol/L, mean ± SD	4.0 ± 1.2	4.2 ± 1.3	t = -0.622	0.536
Creatinine, mg/dL, median (IQR)	33.0 (26.3-40.2)	33.7 (28.7-42.5)	U = 593.00	0.457
Potassium (K), mmol/L, mean ± SD	4.3 ± 0.5	4.5 ± 0.6	t = -1.866	0.066
Sodium (Na), mmol/L, median (IQR)	139.0 (136.0-140.0)	138.0 (138.0-140.0)	U= 618.50	0.742
Creatine kinase, U/L, mean ± SD	3445.2 ± 1905.6	3540.6 ± 2343.3	t = -0.076	0.941
C-reactive protein, mg/L, median (IQR)	12.1 (4.0-43.0)	4.7 (4.0-19.2)	U = 502.50	0.247
Haptoglobin, mg/dL, mean ± SD	11.9 ± 1.0	12.2 ± 1.4	t = -0.974	0.333

Outcomes, impressions, and complications

Hospital admission was required in 51 patients (53.1%), with a mean length of stay of 2.86 days (SD: 2.12). Admission occurred in 31 of 65 children with influenza A (47.7%) and 20 of 31 children with influenza B (64.5%), but this difference was not statistically significant (p = 0.122). Only one child required pediatric ICU (PICU) admission, and the child belonged to the influenza B group. Treatment with oseltamivir (Tamiflu) was given to 89 patients (92.7%). Table 3 presents the complications observed in patients with influenza A and influenza B. Febrile seizures were reported in three patients with influenza A and one patient with influenza B. Hypersensitivity reaction, neutropenia, and thrombocytopenia were observed only in patients with influenza A, while photophobia and stridor were reported exclusively in patients with influenza B. The overall low incidence and distribution of these complications suggest that there was no significant difference in complication rates between the two influenza types (p = 0.350).

**Table 3:** Distribution of complications among patients with influenza A and influenza B Percentages for influenza A and influenza B are calculated within each influenza group among patients who developed complications (influenza A, n = 8; influenza B, n = 3). Percentages in the Total column are based on all patients with complications (n = 11)

Complication	Influenza A (n = 8), n (%)	Influenza B (n = 3), n (%)	Total (n = 11), n (%)
Febrile seizures	3 (37.5%)	1 (33.3%)	4 (36.4%)
Hypersensitivity reaction	1 (12.5%)	0 (0.0%)	1 (9.1%)
Neutropenia	2 (25.0%)	0 (0.0%)	2 (18.2%)
Photophobia	0 (0.0%)	1 (33.3%)	1 (9.1%)
Stridor	0 (0.0%)	1 (33.3%)	1 (9.1%)
Thrombocytopenia	2 (25.0%)	0 (0.0%)	2 (18.2%)

## Discussion

Our study evaluated the clinical presentation, laboratory characteristics, and outcomes of pediatric patients with influenza A and influenza B infections in Bahrain, with a particular focus on influenza-associated myositis. Calf pain and refusal to walk were significantly more common among children with influenza B infection than among those with influenza A infection. Laboratory parameters, hospitalization rates, length of hospital stay, and complication profiles were largely comparable between the two influenza types. This represents one of the few studies from the Arabian Gulf region examining influenza-associated myositis in children and is the first report to describe this association in Bahrain.

Influenza is a common cause of seasonal respiratory illness in children; however, its extra-respiratory manifestations, particularly benign acute childhood myositis (BACM), remain underrecognized. Calf pain was reported in 13 patients (13.5%), while 12 patients (12.5%) presented with refusal to walk. These findings emphasize that musculoskeletal symptoms are not uncommon during pediatric influenza infection and may represent an important cause of acute gait disturbance in children. Previous studies have demonstrated that BACM typically presents during the recovery phase of influenza infection with bilateral calf pain, gait abnormalities, and transient elevations in CK levels [9-11]. Brisca et al. reported gait abnormalities as the primary reason for emergency department presentation in 87 children (77%) with BACM, highlighting the importance of recognizing this entity to avoid unnecessary neurological investigations [4]. Similar observations were reported by Jiang et al. in a recent cohort of 118 children with influenza-associated myositis, where lower limb pain, calf tenderness, difficulty walking, and elevated CK levels were the predominant presenting features [3].

Our study demonstrated a higher prevalence of calf pain and refusal to walk in children with influenza B infection than in those with influenza A infection. This observation is consistent with several previous reports identifying influenza B as the viral subtype most frequently associated with BACM [10-12]. Although the exact mechanism remains uncertain, influenza B has long been recognized as the viral subtype most commonly implicated in pediatric myositis outbreaks. Although the exact pathophysiology remains incompletely understood, proposed mechanisms include direct viral invasion of muscle tissue, immune-mediated muscle injury, and cytokine-induced inflammatory responses in muscle, resulting in transient muscle necrosis and edema [9,10,13].

Experimental studies have demonstrated that influenza viruses can infect skeletal muscle cells, leading to localized inflammation and muscle fiber necrosis. The greater frequency of myositis observed with influenza B may reflect differences in viral tropism or host immune responses, although further mechanistic studies are needed to better understand this relationship. The significantly higher frequency of calf pain and refusal to walk among children with influenza B in our cohort further supports previous evidence suggesting that influenza B may have a greater propensity for skeletal muscle involvement than influenza A, despite similar overall disease severity and laboratory profiles [11,14].

No statistically significant differences were observed in inflammatory markers, hematological parameters, renal function tests, liver enzymes, or creatine kinase levels. Interestingly, both groups demonstrated substantial elevations in creatine kinase concentrations, supporting the presence of muscle involvement regardless of influenza subtype. These findings suggest that while influenza B may be more likely to present clinically with overt manifestations of myositis, the biochemical extent of muscle injury may not differ substantially between influenza A and B infections. Similar observations have been reported in previous pediatric studies, where marked elevations in creatine kinase were frequently observed despite the generally benign clinical course and favorable outcomes of affected children [1,5,9,10].

An additional noteworthy finding was the marked elevation of CK levels observed in both influenza A and influenza B groups, with mean values exceeding 3,400 U/L. Elevated CK is a well-recognized laboratory hallmark of influenza-associated myositis and reflects transient skeletal muscle injury. Previous studies have reported substantial CK elevations in affected children, frequently ranging from several hundred to several thousand units per liter. Jiang et al. reported significantly elevated CK concentrations among children with influenza A-associated myositis, while Attaianese et al. and Capoferri et al. similarly identified CK elevation as the most consistent laboratory abnormality in benign acute childhood myositis [3,9,10]. Despite the prominent CK elevation observed in our cohort, clinical outcomes remained favorable, and no significant renal complications were identified, consistent with observations from previous studies [1,5,14].

The demographic characteristics observed in this study are generally consistent with existing literature. Male patients accounted for nearly 60% of cases, supporting previous reports demonstrating a male predominance among children with influenza-associated myositis. Several cohorts have reported male proportions ranging from 60% to 75%, although the underlying mechanism remains unclear and may involve genetic, hormonal, or immunological factors [1,3,9]. The mean age of approximately four years in our cohort is slightly younger than that reported in other studies of benign acute childhood myositis, which generally describe a peak presentation in school-aged children, particularly between six and nine years of age [3,9,10,14]. This difference may reflect regional epidemiological variations, differences in healthcare utilization patterns, referral bias, or the inclusion of younger children in our study.

The overall clinical course was favorable. Although more than half of the patients required hospitalization, the average duration of admission was relatively short, and only one child required pediatric intensive care unit admission. Furthermore, complication rates were low and did not differ significantly between influenza A and influenza B infections. This is consistent with previous studies demonstrating that influenza-associated myositis is generally a self-limiting condition with an excellent prognosis, characterized by rapid clinical improvement and normalization of muscle enzyme levels within days to weeks [1,5,9]. The absence of significant differences in hospitalization rates and complications between influenza subtypes suggests that the increased frequency of myositis manifestations observed in influenza B does not necessarily translate into greater overall disease severity.

An important clinical implication of these findings is the need for heightened awareness among emergency physicians and pediatricians when evaluating children presenting with acute lower limb pain or refusal to walk during influenza season. Such presentations may raise concern for serious neurological, orthopedic, or rheumatological conditions, often leading to extensive diagnostic evaluations. Recognition of influenza-associated myositis as a relatively common and generally benign complication may facilitate timely diagnosis, reduce unnecessary investigations, and provide reassurance to families. In regions with seasonal influenza circulation, influenza testing should be considered in children presenting with otherwise unexplained calf pain or gait disturbances.

The present study has several strengths. It represents one of the few studies from the Middle East evaluating influenza-associated myositis and provides valuable regional data regarding the clinical differences between influenza A and influenza B infections. In addition, laboratory and outcome data were systematically analyzed, allowing a comprehensive comparison between the two viral subtypes. Several limitations should be acknowledged. The retrospective design carries an inherent risk of incomplete documentation and missing data. The study was conducted at a single tertiary care center, which may limit generalizability. Furthermore, serial creatine kinase measurements were not consistently available, preventing assessment of the temporal evolution of muscle injury. The modest sample size may also have reduced the ability to detect smaller differences in uncommon complications and clinical outcomes.

Future multicenter prospective studies involving larger pediatric populations are warranted to better define the epidemiology, pathophysiology, and predictors of influenza-associated myositis. Such studies may also clarify whether specific viral strains, host characteristics, or immunological factors contribute to the increased propensity for myositis observed among children with influenza B infection.

## Conclusions

Influenza-associated myositis is an underrecognized manifestation of pediatric influenza, with influenza B associated with significantly higher rates of calf pain and refusal to walk than influenza A. However, overall hospitalization rates, complication rates, and CK elevations were comparable between the two subtypes. These findings highlight the importance of considering influenza-associated myositis in children presenting with acute gait disturbances during influenza season. Early recognition may facilitate timely diagnosis, avoid unnecessary neurological and orthopedic investigations, reduce healthcare utilization, and provide reassurance regarding the generally favorable prognosis of this condition. Larger prospective multicenter studies are needed to further characterize the epidemiology, pathophysiology, and predictors of influenza-associated myositis in children.
